# Working Memory Network Changes in ALS: An fMRI Study

**DOI:** 10.3389/fnins.2016.00158

**Published:** 2016-04-19

**Authors:** Anne-Katrin Vellage, Maria Veit, Xenia Kobeleva, Susanne Petri, Stefan Vielhaber, Notger G. Müller

**Affiliations:** ^1^Neuroprotection Group, German Centre of Neurodegenerative DiseasesMagdeburg, Germany; ^2^Berlin School of Mind and Brain, Humboldt-UniversityBerlin, Germany; ^3^Department of Neurology and Clinical Neurophysiology, Medical SchoolHannover, Germany; ^4^Department of Neurology, Otto-von-Guericke UniversityMagdeburg, Germany

**Keywords:** working memory, fMRI, ALS, prefrontal cortex, selective attention

## Abstract

We used amyotrophic lateral sclerosis (ALS) as a model of prefrontal dysfunction in order to re-assess the potential neuronal substrates of two sub processes of working memory, namely information storage and filtering. To date it is unclear which exact neuronal networks sustain these two processes and the prefrontal cortex was suggested to play a crucial role both for filtering out of irrelevant information and for the storage of relevant information in memory. Other research has attributed information storage to more posterior brain regions, including the parietal cortex and stressed the role of subcortical areas in information filtering. We studied 14 patients suffering from ALS and the same number of healthy controls in an fMRI-task that allowed calculating separate storage and filtering scores. A brain volume analysis confirmed prefrontal atrophy in the patient group. Regarding their performance in the working memory task, we observed a trend toward slightly impaired storage capabilities whereas filtering appeared completely intact. Despite the rather subtle behavioral deficits we observed marked changes in neuronal activity associated with ALS: Compared to healthy controls patients showed significantly reduced hemodynamic responses in the left occipital cortex and right prefrontal cortex in the storage contrast. The filter contrast on the other hand revealed a relative hyperactivation in the superior frontal gyrus of the ALS patients. This hyperactivation might reflect a possible compensational mechanism for the prefrontal degeneration found in ALS. The reduced hemodynamic responses in the storage contrast might reflect a disruption of prefrontal top-down control of posterior brain regions, a process which was especially relevant in the most difficult high load memory task. Taken together, the present study demonstrates marked neurophysiological changes in ALS patients compared to healthy controls during the filtering and storage of information in spite of largely intact behavior. With respect to the neuronal substrates of the two working memory processes under investigation here, the results suggest that it is rather the degree to which top-down control is required for task completion that determines prefrontal cortex involvement than the specific nature of the process, i.e., storage vs. filtering.

## Introduction

Working memory (WM) refers to the process of maintaining and modulating information for a short amount of time (Baddeley, 1986). Because the information that can be actively stored is limited (Cowan, 2001) irrelevant information has to be filtered out first. Selective attention fulfills this role by directing the focus on information that is relevant for the task at hand. In the last decades a large body of research has been dedicated to the investigation of the neural mechanisms underlying WM and selective attention (Corbetta and Shulman, 2002; Todd and Marois, 2005; Rottschy et al., 2012). The results of several lesion and single cell studies pointed to the prefrontal cortex (PFC) as an eligible candidate for the storage of information (Funahashi et al., 1993; Chafee and Goldman-Rakic, 1998). In addition to the PFC, regions in the parietal and temporal cortex were found to discharge even after withdrawal of visual stimuli (Miller and Desimone, 1994; Chafee and Goldman-Rakic, 1998). Within these areas a functional segregation into a dorsal space-based pathway and a ventral object-based pathway was proposed (Goldman-Rakic, 1987). With the goal of identifying a core network underlying WM, a meta-analysis over several studies was conducted by Rottschy et al. (2012). The reported core network consisted of the bilateral middle frontal gyrus (MFG), the inferior frontal gyrus (IFG), the insulae, the lateral PFC, and the inferior parietal cortex (IPC). Whereas the parietal cortex is sensitive to memory load (Todd and Marois, 2005; Xu and Chun, 2005), the PFC is not. Hence, it has been suggested that the PFC is rather involved in top-down control than storage *per se* (e.g., Corbetta and Shulman, 2002; Müller and Knight, 2006). The latter process seems more related to the load sensitive parietal cortex, which also determines the WM limit (e.g., Vogel and Machizawa, 2004; Todd and Marois, 2005; McNab and Klingberg, 2007). However, whereas this hypothesis is underpinned by a study using multi voxel pattern analysis (Christophel et al., 2012), the authors of a second study using the same method did not find evidence for the parietal cortex coding memorized information (Riggall and Postle, 2012) and others have suggested that this task is rather accomplished by extrastriate visual areas (e.g., Becke et al., 2015). Therefore, findings considering the exact neural correlates of WM storage are still equivocal.

The PFC has been postulated as a crucial brain region for information filtering, too. But again, also the inferior and superior parietal cortices (IPC and SPC) have been suggested to take part in top-down attentional control of bottom-up visual processing. The latter has been attributed to a ventral network including the temporo-parietal junction, the insulae, inferior, and MFG, the IPC as well as the frontal eye fields (Corbetta and Shulman, 2002). In sum, WM and selective attention seem to rely on similar brain regions whereby both parietal and frontal regions were identified as essential nodes for the two processes. However, the exact division of labor between those regions in supporting the named sub-processes of WM remains unclear.

Neurological diseases such as Parkinson's and Alzheimer's which are characterized by the degeneration of certain brain regions have been used previously for making inferences about the affected brain regions' contribution to WM and attention (Finke et al., 2013; Blatt et al., 2014). With regard to the PFC, ALS can be considered a potential model (Iwanaga et al., 1997). ALS is characterized by the deterioration of mainly motor neurons but also other frontal neurons (Abrahams et al., 1996) as well as whiter matter fibers (Abrahams et al., 2005; Lillo et al., 2012; Hartung et al., 2014). The disease is characterized clinically by progressive muscle weaknesses. Hence, until recently most research has focused on the motor system, whereas the investigation of cognitive deficits in ALS patients was foregrounded in the last years (Phukan et al., 2012; Elamin et al., 2013). Following recent consensus criteria (Strong et al., 2009) a type of ALS with additional cognitive and/or behavioral impairments can be distinguished from ALS with motor degeneration only. When present, cognitive deficits in ALS patients mainly consist in executive dysfunctions (Abe et al., 1997).

Here, in an attempt to further clarify the contribution of PFC to WM subprocesses, we tested ALS patients' abilities to select and store information in WM. We designed a WM task that allowed separating filtering and storage processes both on a behavioral and a neuronal level. In order to guarantee that the patients were able to solve the demanding WM task we selected ALS patients without obvious cognitive impairment as documented in a neuropsychological test battery. Hence, we did not expect a marked deficit in our task but were rather interested in compensatory neuroplasticity in response to prefrontal pathology. In order to confirm the latter we also assessed the frontal brain volumes—of both gray and white matter—of our participants.

## Materials and methods

### Experimental design

ALS patients and healthy controls performed a WM task in a 3T MRI scanner. Before participating in the main experiment, all subjects completed a shortened version of the WM task outside the scanner.

### Participants

Prior to the experiment all candidates underwent a neuropsychological screening consisting of the Montreal Cognitive Assessment (Costas et al., 2014) and a German translated version of the Edinburgh Cognitive and Behavioral ALS Screen (ECAS; Lulé et al., 2015). To estimate the general intelligence, a vocabulary test (Wortschatztest, (WST), Schmidt and Metzler, 1992) was applied. Additionally, all subjects completed a shortened version of the WM task outside the scanner and only those who performed above 50% in this practice session took part in the later fMRI experiment.

These eligibility criteria allowed us to include 14 (6 female) patients at the average age of 57.00 years (range 43–72 years). They were recruited from the outpatient clinics of the Departments of Neurology at the Medical School of the Otto-von-Guericke University Magdeburg and at the Medical School, Hannover. Following the revised El-Escorial-Criteria (Brooks et al., 2000) one patient was diagnosed with definitive clinical ALS, five patients with probable clinical ALS and eight patients with possible clinical ALS. The revised ALS Functional Rating Scale (ALSFRS-R; Cedarbaum et al., 1999) was used to assess the severity of the disease. The patients had an average ALSFRS-R score of 40.29 (range 32–47). The disease duration was 12.42 (± 4.13 SEM) months on average. Fourteen control participants (8 female) at the age of 58.36 years (range 45–77 years) without a prior history of neurological or psychiatric illness were recruited as controls and were matched for age and education years (Table 1). They received the same cognitive screening procedure and had to fulfill the same inclusion criteria as the patients. All participants gave written informed consent before participation. The study was approved by the local ethics committee.

**Table 1:** **Demographical and neuropsychological data among ALS patients and controls**.

	**Controls (SEM)**	**Patients (SEM)**	**Significance (*t*-test)**
Age (years)	58.36 (9.70)	57.00 (9.10)	0.731
Education (years)	14.07 (2.30)	15.14 (2.74)	0.273
MoCA	27.64 (1.08)	26.21 (2.81)	0.094
ECAS non ALS specific	29.09 (0.90)	29.57 (1.10)	0.748
ECAS ALS specific	78.09 (1.92)	79.07 (2.72)	0.771
ECAS Total score	107.18 (2.24)	108.71 (3.49)	0.715
WST	31.79 (2.42)	32.71 (3.95)	0.462

ALS, Amyotrophic lateral sclerosis; ECAS, Edinburgh Cognitive and Behavioral ALS Screen; MoCA, Montreal Cognitive Assessment; WST, Wortschatztest.

### Working memory task

**Figure 1:**
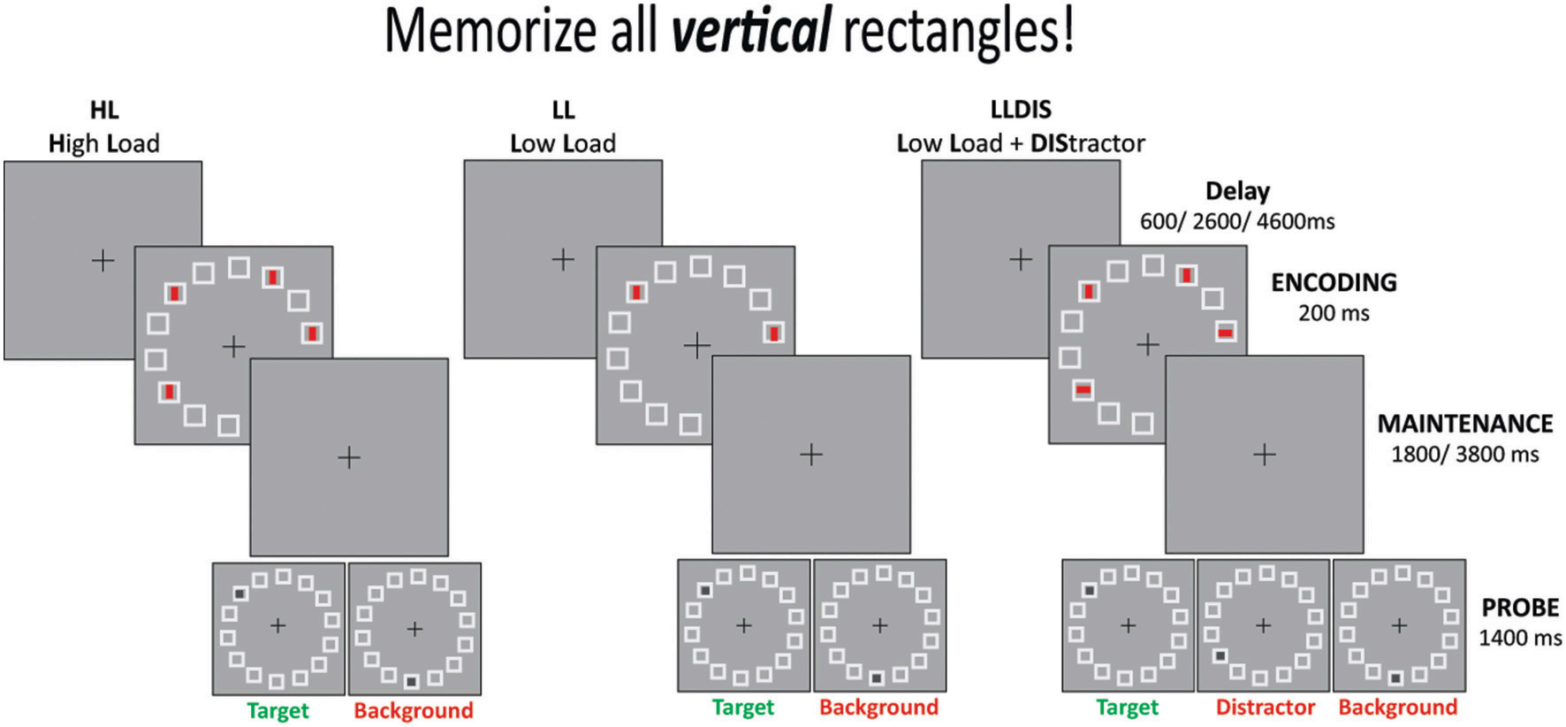
**Schematic illustration of the experimental design**.

The fMRI experiment (Figure 1) included three conditions with the following demands: high memory storage (high load, HL), low memory storage (low load, LL) and low memory but high filtering (low load + distractors, LLDIS). Participants were instructed to memorize the vertical rectangles only. The HL condition consisted of a memory array with four vertical rectangles, whereas the LL condition consisted of two vertical rectangles only. In the LLDIS condition two vertical rectangles were presented alongside two horizontal rectangles which served as distractors. All stimuli had the same color (red) and were presented within 14 placeholder squares that were arranged in a circle. The memory array was followed by a delay and then by a probe stimulus (gray dot) to which subjects had to decide by button press with the index or middle finger of their right hand whether the probe location had been occupied by a target stimulus in the preceding memory array or not. When the probe stimulus was not in the position of a target, it was either on a position adjacent to the target that was formerly an empty placeholder square or, in case distractors had been presented, with equal probability on a distractor position. The required responses (yes or no) were distributed evenly across all trials. Subjects completed six runs à 60 trials (360 trials in total) with one run lasting 8 min. Conditions were presented in a randomized order.

Stimuli were presented against a gray background (luminance 41.2 cd/m^2^). During the whole experiment a fixation cross was presented in the center of the screen (16.4° from side, 18.8° from top). Memory (size 0.8° × 0.3°, luminance 31 cd/m^2^) and probe stimuli (size 0.3° × 0.3°) appeared within 14 placeholder squares (size 0.9° × 0.9°) arranged in a circle (diameter 7.3°, minimum difference squares 1.5° center to center). The memory array was presented for 0.2 s and was followed by a delay of 1.8 or 3.8 s. All trials ended with a probe stimulus that appeared for 1.4 s and was followed by a delay of 0.6, 2.6, or 4.6 s.

### Behavioral data analysis

For analysis of the behavioral data we first calculated scores for filtering and storage, respectively. This was done by subtracting the percentage correct of the HL condition from percentage correct in the LL condition (storage score) and accuracy measures of the LLDIS condition from the LL condition (filter score; Blatt et al., 2014). The advantage of the scores over raw data (hit rates) is that a potential overall deficit is canceled out by this subtraction method.

### fMRI data acquisition

A 3T MR scanner (Siemens Magnetom Verio syngo MR B19, Erlangen, Germany) equipped with a 32-channel head coil was used to measure blood oxygenation level-dependent (BOLD) brain activity. Stimuli were back-projected by an LCD projector onto a screen positioned behind the coil. The screen was viewed by the subjects via a mirror attached to the head coil. Functional images were acquired with a T2^*^-weighted echo planar imaging (EPI) gradient echo sequence (FoV 224 × 224 mm, voxel size = 3.5 × 3.5 × 3.5 mm, TR = 2000 ms, TE = 38 ms, flip angle = 80°) in an odd-even interleaved sequence. Thirty-two 3.5 mm thick axial slices (64 × 64 mm in plane, no gap) parallel to the AC-PC line were acquired for 255 volumes in each run. Whole-head T1-weighted images were collected with an MP-RAGE sequence (96 sagittal slices, thickness = 2 mm, FoV 256 × 256 mm, no gap, spatial resolution = 1 × 1 × 2 mm, TR = 1660 ms, TE = 5.05 ms, TI = 1100 ms).

### MRI data analysis

Whole-head T1-weighted images were analyzed using the SPM8 software package (Welcome Department of Cognitive Neurology, University College London, UK) and MATLAB R2009b (The Mathwork Inc.), which included segmenting of images in gray matter, white matter, and cerebrospinal fluid. A mask image of the frontal lobe was generated using the WFU PickAtlas (Maldjian et al., 2003, 2004) and coregistered on the T1-images. Volumes were read out for each participant and frontal lobe volumes were separated into gray matter and white matter volumes. These values were corrected for the total brain volume (TBV) by dividing the individual frontal lobe volumes by the individual TBV (corrFLV). Differences in corrFLV between groups were assessed by carrying out a univariate ANOVA with the between-subject factor group.

### fMRI data analysis

Data were processed with the SPM8 software package (Welcome Department of Cognitive Neurology, University College London, UK) and MATLAB R2009b (The Mathwork Inc.), which included slice time correction, realignment to the first volume, co-registration to the individual anatomical images, normalization to the Montreal Neurological Institute (MNI) template (Friston et al., 1995) and resampling into a voxel size of 3 × 3 × 3 mm^3^. Spatial normalized images were smoothed with an isotropic 6 mm FWHM Gaussian kernel and high pass filtered (cut-off 128 s). Global scaling was applied across an individual session to remove global signal drifts before GLM analysis. No subjects had to be excluded due to excessive head motion (more than 5 mm).

BOLD responses were modeled by delta functions at the time of stimulus onsets. For each individual, the time courses of the hemodynamic BOLD responses in the different conditions (HL, LL, LLDIS) were analyzed at the voxel level using a linear regression model that yielded separate time courses for the encoding and response phase of each condition. The movement parameters derived from the realignment process were included as covariates into the model as well as all trials in which the subjects made a wrong response leading to 11 regressors in total for each run (3 × encoding phase: HL, LL, LLDIS; 1 × response phase: merged (HL, LL, LLDIS); 1 × errors; 6 × movement). To identify regions showing filter and memory related BOLD response differences we calculated different contrasts for each subject and each session individually for each condition in the encoding phase in a first-level analysis. To assess memory storage related activity, BOLD responses in the HL condition were contrasted with BOLD responses from the LL condition. This contrast is referred to as “storage contrast.” Similarly the contrast between LLDIS and LL is referred to as “filter contrast.” Then, to look at filter and storage contrasts for each group separately a one sample *t*-test was conducted first on a second-level with the individual contrast images including whole head gray and white matter volumes as covariates. Second, the contrast images of every participant from the first level were subjected to a second-level two sample *t*-test to see differences in filter and storage contrast between groups. In a next step, we calculated ROI analyses for the clusters obtained in the calculated contrasts between groups by using ROIs from the group difference maps. The minimal distance between cluster peaks was set at 18 mm. The ROIs were identified from the group activation maps and were defined at a threshold level of p (uncorr.) 0.001 like in previous studies (Groussard et al., 2010; van de Sand et al., 2015). A small/cluster volume correction was not applied. Beta-values of the fMRI effect-of-interest contrasts were compared between groups by means of a multivariate ANOVA with the between-subject factor group.

### Statistical analysis

Statistical analyses were carried out using the Statistical Package for the Social Sciences v21.0 (SPSS). Behavioral data were analyzed using repeated measures ANOVAS with the between subject factor group (patient, controls) and the within subject factor score (filter score, memory score). In case of significant interaction or main effects, *post hoc t*-tests were carried out. Differences in corrFLV between groups were assessed by carrying out a univariate ANOVA with the between-subject factor group.

## Results

### Behavioral results

**Figure 2:**
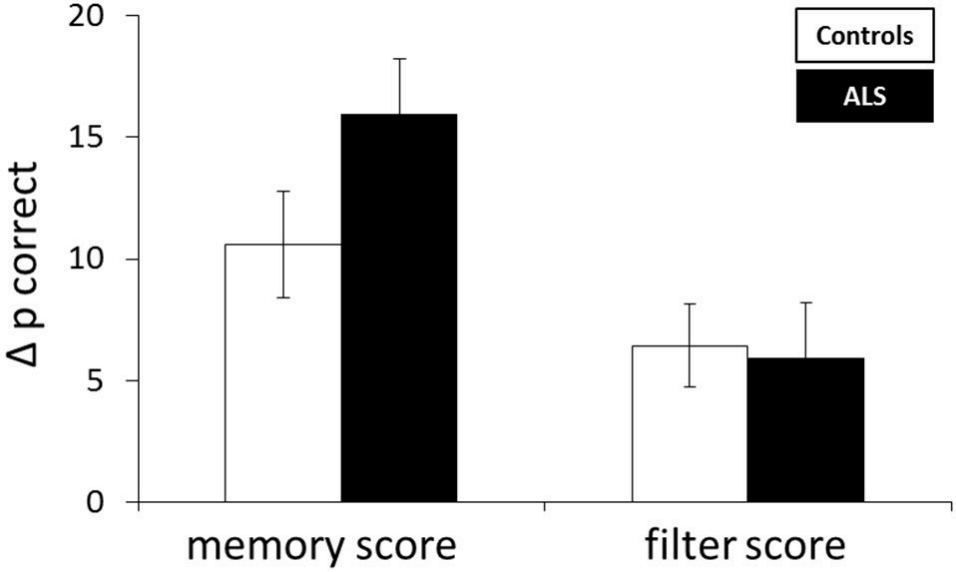
**Mean difference in accuracy (Δp correct) reflecting storage and filter score**. Error bars indicate the standard error of the mean.

Storage and filter scores were calculated from accuracy measures. Differences between performance in the LL and HL condition were referred to as storage score with large values indicating impairment with increasing memory load, i.e., a storage deficit. The filter score was assessed by calculating the difference between the LL and the LLDIS condition. In the case of distractors being unnecessarily stored (filtering deficit), performance in the LLDIS condition should be low leading to a higher filter score. An rANOVA with the within factor score (storage, filter score) and the between factor group (patient, controls) revealed a significant main effect of score [*F*_(1, 26)_ = 17.363, *p* < 0.001] and a trend toward a group × score interaction (*F*_(1, 26)_ = 2.942, *p* = 0.098). In general, storage scores were higher than filter scores (*p* < 0.001). Means and standard errors of the mean for memory and filter scores of both groups can be depicted from Table 2; Figure 2.

**Table 2:** **Means and standard error of the mean (SEM) for storage and filter scores in the working memory task**.

		**Storage score**	**Filter score**
Controls	Mean (SEM)	10.60 (2.20)	6.43 (1.71)
ALS	Mean (SEM)	15.95 (2.27)	5.95 (2.24)

ALS, Amyotrophic lateral sclerosis.

### MRI results

**Figure 3:**
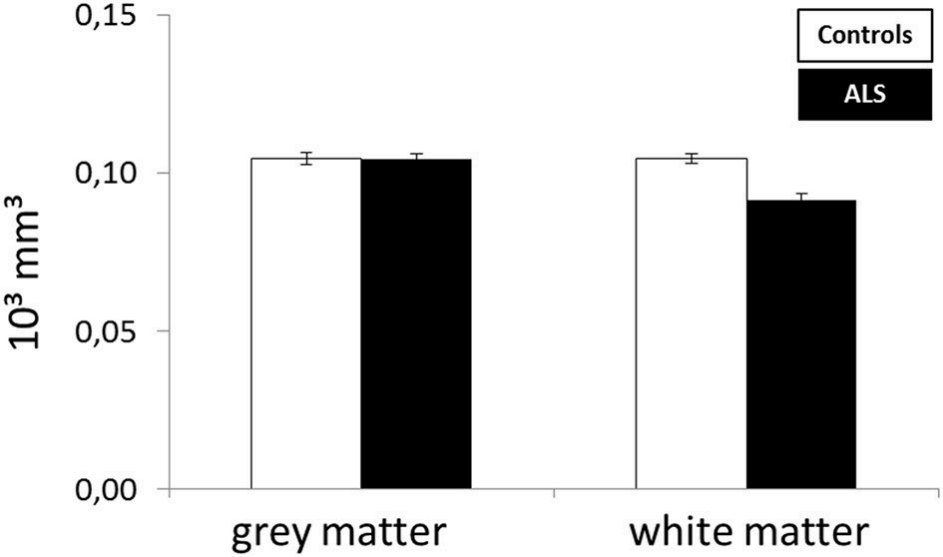
**Difference in corrected frontal lobe volume between ALS patients and healthy controls**.

To assess the frontal pathology in ALS patients, corrFLV of gray and white matter were compared between patients and controls by means of a univariate ANOVA (Figure 3). A significant higher corrFLV in white matter was found in controls compared to ALS patients [*F*_(1, 27)_ = 24.528, *p* < 0.001] whereas no significant difference was found in gray matter volume between groups [*F*_(1, 27)_ = 0.347, *p* = 0.561].

### fMRI results

**Figure 4:**
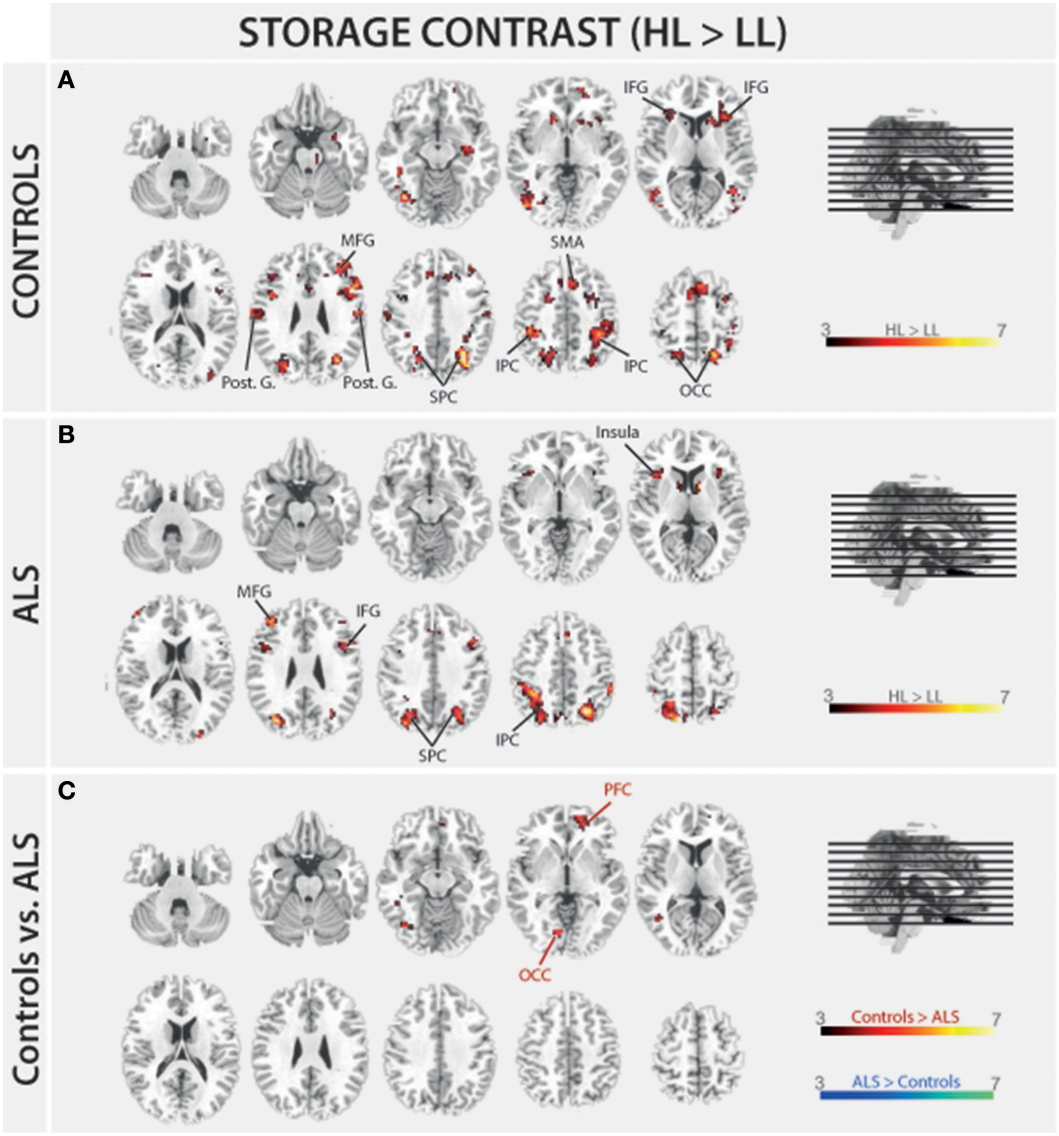
**Task-related changes in BOLD signal during encoding: The color bar indicates the *T*-value; (A)** Control group activation map for the storage contrast (HL > LL); **(B)** ALS group activation map for the storage contrast (HL > LL); **(C)** Group activation map for the difference in storage contrast between ALS and control group (red/yellow: Controls > ALS, blue/green: ALS > Controls).

**Figure 5:**
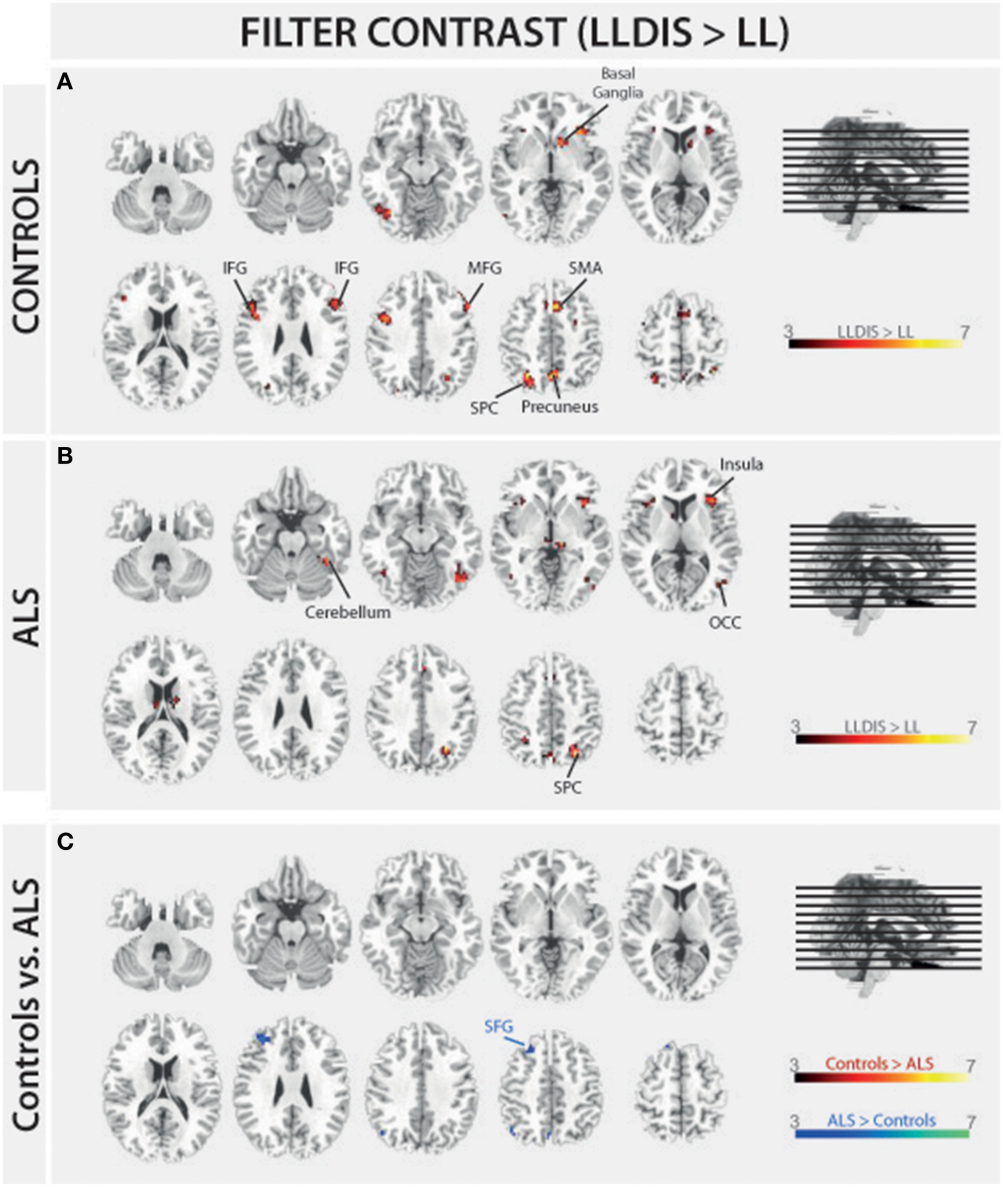
**Task-related changes in BOLD signal during encoding: The color bar indicates the *T*-value; (A)** Control group activation map for the filter contrast (LLDIS > LL); **(B)** ALS group activation map for the filter contrast (LLDIS > LL); **(C)** Group activation map for the difference in filter contrast between ALS and control group (red/yellow: Controls > ALS, blue/green: ALS > Controls).

In order to address BOLD differences between ALS patients and healthy controls we compared fMRI data of the filter and storage contrasts across both groups. In control subjects several brain regions emerged during the storage contrast on a threshold level of 0.001 uncorr. including bilateral superior (SPC) and inferior (IPC) parietal cortex, postcentral gyrus, right inferior frontal gyrus (IFG), left occipital cortex (OCC), right insula, right MFG, and supplementary motor area (SMA). In ALS patients a reduced net of activation during the storage contrast was found on the same threshold level including bilateral SPC, right IFG, left MFG, left insula, and left IPC (Figure 4). Similarly, in the filter contrast more brain regions were found to be activated in controls than in ALS patients. Whereas ALS patients recruited the right SPC, the right cerebellum, the right OCC, and right insula only during filtering, controls recruited bilateral IFG, precuneus, SMA, right MFG, left SPC, and right basal ganglia (caudate nuclei). Peaks of all clusters are reported in Table 3 and are displayed in Figures 4, 5A,B).

**Table 3:** **Peak activations for the storage and filter contrasts of controls and ALS patients**.

**Anatomical structure**	**Hemisphere**	**MNI coordinates (x, y, z)**			**Max. *T*-value**	**Cluster size**
**Controls: HL > LL**						
SPC/OCC	R	30	−67	31	8.83	95
	R	18	−61	58	7.01	42
	R	54	−70	1	5.10	10
	L	−21	−70	40	6.94	27
	L	−21	−61	58	5.14	13
	L	−39	−70	−11	7.41	69
	L	−27	−76	25	4.99	13
IPC	R	36	−40	46	5.83	46
	L	−39	−40	49	6.02	40
IFG	R	48	11	25	5.94	55
Insula	R	33	20	1	5.72	21
SMA	R	3	17	52	5.68	36
MFG	R	36	35	31	5.49	25
Postcentral gyrus	R	54	−19	28	5.31	12
	L	−63	−22	28	5.15	14
**ALS: HL > LL**						
SPC	R	24	−64	46	7.11	123
		30	−73	31	4.82	
	L	−33	−79	31	9.45	130
		−21	−76	52	6.61	
		−24	−55	55	4.95	12
IPC	L	−36	−46	49	5.85	62
IFG	R	45	5	28	6.89	45
Insula	L	−39	17	1	4.80	10
MFG	L	−39	35	25	5.55	20
**CONTROLS: LLDIS > LL**						
SPC	L	−27	−64	49	6.27	31
Precuneus		0	−64	46	6.70	24
IFG	R	30	23	−5	5.59	13
	L	−39	2	34	5.40	31
SMA	R	3	14	49	6.30	38
MFG	R	48	23	28	5.18	34
Basal Ganglia (Caudate ncl.)	R	9	8	−2	6.13	15
**ALS: LLDIS > LL**						
SPC	R	27	−67	46	7.88	44
OCC	R	45	−70	−14	5.16	19
Insula	R	36	20	1	6.55	23
Cerebellum	R	36	−46	−29	5.59	11

L, left; R, right; ALS, Amyotrophic lateral sclerosis; HL, high load; IFG, inferior frontal gyrus; IPC, inferior parietal cortex; LL, low load; LLDIS, low load + distractor; MFG, middle frontal gyrus; OCC, occipital cortex; SMA, supplementary motor area; SPC, superior parietal cortex.

In a second step filter and storage contrasts were compared between groups. Compared to ALS patients controls recruited left OCC [*F*_(1, 26)_ = 25.834, *p* < 0.000] and right PFC [*F*_(1, 26)_ = 23.994, *p* < 0.000] more during the storage of information. ALS patients showed no stronger signal in storage contrast compared to controls at a threshold of *p* = 0.001 (uncorrected).

During filtering no BOLD responses above threshold were found in controls compared to ALS patients. However, ALS patients showed a stronger activation in left superior frontal gyrus (SFG, Cluster 1: [*F*_(1, 26)_ = 26.648, *p* < 0.000], Cluster 2: [*F*_(1, 26)_ = 8.167, *p* = 0.008] during filtering compared to controls. Peaks of all clusters are reported in Table 4.

**Table 4:** **Peak activations for the difference in storage and filtercontrasts between groups**.

**Anatomical structure**	**Hemisphere**	**MNI coordinates (x, y, z)**			**Max. *T*-Value**	**Cluster size**
**HL > LL: Controls > ALS**						
OCC	L	−12	−79	−8	5.03	16
PFC	R	15	50	−5	4.66	34
**HL > LL: ALS > Controls**						
−	−	−	−	−	−	−
**LLDIS > LL: Controls > ALS**						
−	−	−	−	−	−	−
**LLDIS > LL: ALS > Controls**						
SFG	L	−33	35	22	4.46	16
	L	−27	29	49	4.30	11

L, left; R, right; ALS, Amyotrophic lateral sclerosis; HL, high load; LL, low load; LLDIS, low load + distractor; OCC, occipital cortex; PFC, prefrontal cortex; SFG, superior frontal gyrus.

## Discussion

The present study aimed at investigating the neuronal basis of selective filtering and information storage within WM. These processes were investigated in ALS patients because of their known prefrontal deficit. The PFC has been suggested to play a major role both in filtering and storage (Chafee and Goldman-Rakic, 1998; Corbetta and Shulman, 2002). Patients and healthy controls had to perform a task in which storage and filter demands were manipulated. In the baseline condition participants had to memorize two items only. The ability to effectively store information was assessed by increasing memory load to four items; the ability to filter out irrelevant information was measured by adding two distractors to the memory display. From the pairwise comparisons of the two demanding conditions with the baseline condition, storage and filter scores (behavioral data) and contrasts (fMRI data) were derived. Whereas the behavioral filter score was the same in both groups under investigation, a trend toward a relative storage deficit was seen in ALS patients compared to controls. Besides these rather small behavioral group differences we found marked changes in the fMRI data. In the storage contrast of the controls the inferior and MFG, the SMA, the postcentral cortex, the superior and IPC, and the occipital OCC emerged (Figure 4A). ALS patients recruited frontal (IFG and MFG) and parietal (IPC) cortices and the left insula only (Figure 4B). A direct comparison between the storage contrasts of both groups (Figure 4C) confirmed a significant reduction of the hemodynamic response in ALS patients in prefrontal and OCC. The most likely source of this reduced network activity is the proposed ALS-related prefrontal pathology (Agosta et al., 2013) which was confirmed in our sample by means of a brain volume analysis revealing decreased white matter in frontal brain volumes in the patients compared to healthy controls (Figure 3). An ALS-related white matter pathology was reported in other studies as well (Abrahams et al., 2005; Lillo et al., 2012; Hartung et al., 2014) and can likely contribute to cortical activity differences (Lockhart et al., 2015). The frontal alterations in white matter might then have led to an impairment of feedforward and feedback connections between the PFC and posterior areas like the extrastriate visual cortices (Miller and D'Esposito, 2005; Clément and Belleville, 2010). Hence, the reduced network activity in ALS may reflect a disruption of prefrontal top-down control of these posterior brain regions resulting from reduced white matter connectivity. Top-down control was especially required in the high load storage condition which was the most difficult in the present study. Consequently, only in this condition a subtle behavioral deficit was to be observed in the patients. Information filtering was found to be behaviorally intact in the ALS patients. Nevertheless, the fMRI contrast between the distractor and the baseline condition revealed marked activity differences between the groups. In healthy controls the inferior and MFG, the SMA, the SPC, the precuneus and the basal ganglia were found to be associated with filtering (Figure 5A). In contrast, the ALS patients showed activations in the SPC, cerebellum, OCC and the insulae (Figure 5B). The direct group comparison (Figure 5C) then revealed a stronger hemodynamic response in ALS patients in the SFG, whereas relative reductions in hemodynamics were not seen anywhere in the patients' brains. Although counterintuitive at first sight, the finding of frontal hyperactivation in our view is also best explained by the frontal atrophy observed in our ALS patients. Only instead of a breakdown of prefrontal control as seen in the high memory load condition, in the case of filtering the prefrontal structural atrophy was obviously compensated for by hyperactivating the SFG. Such compensational hyperactivation during memory tasks in patients suffering from early-phase neurodegenerative diseases has been described before, for example in the hippocampus of patients with mild cognitive impairment (MCI; Clément and Belleville, 2010) and also in ALS patients (Agosta et al., 2013; Zaehle et al., 2013).

To summarize, the frontal pathology in the group of ALS patients might have led to a reduction of hemodynamic responses in prefrontal, OCC and fusiform gyrus during memory storage and to a hyperactivation of superior frontal brain areas during filtering. The first probably signals an impending breakdown of the storage process, whereas the latter constitutes a compensational mechanism that guaranteed completely intact filtering behavior. Behavioral data also indicated that the high load storage task was more difficult than the filtering task, a confounder that usually cannot be avoided in the type of tasks used here and, therefore, has routinely emerged in prior studies as well (e.g., McNab and Klingberg, 2007; Blatt et al., 2014). Hence, the differences in hemodynamic responses between the filter and the storage contrast may not solely reflect the specific challenges of the two processes but may have been driven by task difficulty, too. That is, the more difficult high storage load task probably required more prefrontal control than the somewhat easier filtering task. From this it follows that the ALS patients could fully compensate for the easier filter task by hyperactivating the PFC but not for the more difficult high storage load task where prefrontal top-down control of posterior brain regions began to decline. More severely affected patients may show such a breakdown of prefrontal control in easier tasks as well. This rises another critical issue, namely that of a positive selection bias.

In the present study only patients without behavioral deficits in a neuropsychological screening procedure and with above chance performance levels in a training version of the WM task were included. Hence, with this selection bias the results cannot be considered representative for all ALS patients as these constitute a rather heterogeneous sample. Indeed, Elamin et al. (2013) have shown in a longitudinal study of 186 ALS patients that patients differ tremendously in their cognitive abilities in the course of the disease. Patients with pronounced cognitive deficits at their initial visit show a fast cognitive decline during follow- up. Patients without cognitive deficits at baseline on the other hand tend to largely maintain their cognitive abilities. This heterogeneity might also explain the rather ambiguous picture in the literature on WM in ALS where no, mild as well as strong impairments have been reported (Ringholz et al., 2005; Volpato et al., 2010; Hammer et al., 2011; Phukan et al., 2012).

Even though the present results may not be considered representative, they clearly show that even at stages with an almost intact behavior, patients suffering from neurodegenerative disorders like ALS demonstrate marked changes on a neurophysiological level. Enhanced hemodynamic responses seem to reflect mechanisms to functionally compensate for the typical ALS pathology in frontal brain structures during less demanding filtering tasks. Reduced neuronal activity occurs when information storage becomes too demanding for the pathologic structural changes to be fully compensated for. Moreover, our results indicate that the PFC is involved in both memory storage and filter processes; and it seems to be the degree to which these processes are challenged that modulates PFC activity rather than the specific nature of the underlying process. In sum, the present data show how dynamic the brain can react when faced with neuronal cell loss.

## Authors contributions

AV, development of paradigm, set up, data acquisition, data analysis, agreement to be accountable for all aspects of the work; MV, substantial contributions to work, data acquisition, data analysis, drafting the paper, final approval, agreement to be accountable for all aspects of the work; XK, SP, substantial contributions to work, patient recruitment, drafting the paper, final approval, agreement to be accountable for all aspects of the work; SV, substantial contributions to work, patient recruitment, data analysis, drafting the paper, final approval, agreement to be accountable for all aspects of the work; NM, substantial contributions to conception or design of work, set up, data analysis, drafting the paper, final approval, agreement to be accountable for all aspects of the work.

## Funding

This work was supported by grants to NM from the Deutsche Forschungsgemeinschaft (DFG Mu1364/4 to NM) and to SV from the Foundation of Medical Research (950037), Frankfurt, Germany.

### Conflict of interest statement

The authors declare that the research was conducted in the absence of any commercial or financial relationships that could be construed as a potential conflict of interest.

## Abbreviations

ALS: Amyotrophic lateral sclerosis
ALSFRS-R: ALS functioning rating scale revised
BOLD: blood oxygen level-dependent
corrFLV: corrected frontal lobe volume
ECAS: Edinburgh Cognitive and Behavioral ALS Screen
fMRI: functional magnetic resonance imaging
HL: high load
IFG: inferior frontal gyrus
IPC: inferior parietal cortex
LL: low load
LLDIS: low load + distractor
MCI: mild cognitive impairment
MFG: middle frontal gyrus
MNI: Montreal Neurological Institute
MoCA: Montreal Cognitive Assessment
MVPA: multivoxel pattern analysis
OCC: occipital cortex
PFC: prefrontal cortex
SMA: supplementary motor area
SPC: superior parietal cortex
TBV: total brain volume
WM: working memory
WST: Wortschatztest.
